# The prevalence and the reasons of issuing permission for therapeutic abortion in department of forensic medicine, Kermanshah, Iran, during 2005 to 2010

**DOI:** 10.1186/s13104-019-4622-4

**Published:** 2019-09-13

**Authors:** Azam Sharifi, Maryam Janatolmakan, Alireza Khatony

**Affiliations:** 1 School of Nahavand Paramedical, Hamadan University of Medical Sciences, Hamadan, Iran; 2 Clinical Research Development Center, Imam Reza Hospital, Kermanshah University of Medical Sciences, Kermanshah, Iran; 3 Health Institute, Social Development and Health Promotion Research Center, Kermanshah University of Medical Sciences, Kermanshah, Iran

**Keywords:** Abortion, Forensic medicine, Permission, Therapeutic abortion

## Abstract

**Objective:**

The present study aimed to investigate the prevalence and the reasons of issuing permission for therapeutic abortion in department of forensic medicine, Kermanshah-Iran.

**Results:**

There were a total number of 428 applications for issuing permits. The most common reasons of issuing permit for therapeutic abortion were fetal and maternal problems, specifically cerebral abnormalities (70.8%), and anencephaly (30.3%). Furthermore, 354 (82/7%) out of 428 applications were able to get the legal permit and 17.3% of the applications did not receive permission, which was mainly due to “the lack of maternal indication”. Increased knowledge of physicians and clinical personnel on indications of therapeutic abortions and related regulations would lead to the implementation of strategies which prevent void referrals to the department of forensic medicine and a better execution of therapeutic abortion law. By improving the health condition of pregnant women who seek pregnancy termination, informing them about indications of therapeutic abortions, and developing proper strategies to make pregnant women more acquainted with legal cases of abortion, we can take a significant step towards helping pregnant women and promoting their health.

## Introduction

Abortion refers to the removal of conception products before 20th week, when the gestational weight is about 500 g [1]. According to the World Health Organization (WHO), unsafe abortion is a method used to terminate unwanted pregnancy by non-experts or in an environment that lacks minimum medical standards or both [2–4]. Today, unsafe abortion is one of the most important global challenges in terms of Public Health and Human Rights [5, 6]. Statistics indicate that, 56% of the abortions in developing countries are done using unsafe methods while only 6% of the abortions in developed countries are done unsafely. However in total, 40% of women at childbearing age are living in the countries where the abortion is banned or is permitted only when the physical and mental health of the mother is in danger [7]. Unsafe abortion is one of the main reasons for death of pregnant women in developing countries [5, 6, 8]. According to the World Health Organization’s statistics, there are 210 million pregnancy cases per year, from which nearly 50 million lead to abortion, 19–20 million of which are done illegally and unsafely outside health centers by non-experts. Based on estimation, 70 thousand women die per year due to complications of unsafe abortions. The death due to unsafe abortion accounts for 13% of total mortality of the pregnant women. However, 97% of the illegal and unsafe abortions happen in developing countries and more than half of them in Asia, which consequently lead to thousands of deaths per year [9–12]. One out of four mothers, who have had unsafe abortion, will most probably develop severe complications due to unsafe abortion and will require hospitalization [13]. There are no exact statistics on illegal abortions in Iran. However, the statistics reported by the health centers are so shocking that has made the development of relevant legislation inevitable in order to facilitate therapeutic abortion [14]. Prior to the Islamic Revolution in 1979, the abortion was legally banned except in cases where the health of mother was in danger, and the related laws had been formulated regardless of Sharia. However, after Islamic Revolution in Iran, the laws regarding abortion were changed according to Sharia [15]. In recent years, the statistics on illegal abortions, mortality rate of pregnant women and fetal abnormalities in the country and also the shortcoming of laws in this regard have caused related organizations including Ministry of Health and Department of Forensic Medicine to pay attention to this issue and discuss it in the research and scientific centers which led to the approval of an act entitled; “therapeutic abortion” in June 2005. According to this law, abortion is legal in cases where the mother’s life is in danger, and also in cases of fetal abnormalities such as anencephaly, or when the pregnancy produces infant with disorders such as major thalassemia or bilateral polycystic kidney disease. However, the cases of therapeutic abortion should be approved by three specialist physicians with the consent of mothers and final acceptance by the office of legal medicine. Legal abortion is only allowed before week 19th of the pregnancy [1].

In general, every condition that threatens the mother’s health and endangers pregnancy is included as the indication for therapeutic abortion [16]. Since the health of mother is the main element of family and society’s health, lack of information among pregnant women about the legal centers for abortion and complications of illegal and unhealthy abortion endangers their health and creates financial problems for health system of the country. Moreover, developing proper strategies and correct planning in this regard seems to be beneficial. Thus, the present study aimed to examine the frequency and reasons of issuing permission for therapeutic abortion in the department of forensic medicine, Kermanshah-Iran, during 2005 to 2010.

## Main text

### Methods

This descriptive study was conducted over 3-month time between May and June 2018. A total of 428 applications of pregnant women who attended the Department of Forensic Medicine in Kermanshah, Iran from April 2005 to March 2010, to get abortion permit were investigated in this study. A researcher-made questionnaire was used to collect data on age, education level and employment status of mother, fetal age, maternal and fetal indications for abortion, and the number of issued and non-issued permits. First, the researchers obtained the necessary permission from the Ethics Committee of Kermanshah University of Medical Sciences and the General Office of Legal Medicine of Kermanshah Province. Later, they attended the Medical Record Department of the General Office of Legal Medicine and examined the applications for therapeutic abortion during 2005–2010 and then completed the questionnaire based on them.

#### Data analysis

Data were analyzed using the Statistical Package for Social Sciences (SPSS v.16.0; SPSS Inc., Chicago, IL, USA) and descriptive statistic (frequency percentage, mean and standard deviation).

#### Ethical considerations

Ethical approval was obtained from the Ethics Committee of Kermanshah University of Medical sciences. Permission was also obtained from the general office of legal medicine of Kermanshah Province-Iran.

### Results

Results of the present study showed that, there were a total of 428 applications for issuing abortion permit during 2005 to 2010. More than 82% of the requests for abortion permit were successful. The average age of the mothers and fetus were 29 ± 7 years and 13 ± 4 weeks, respectively. The minimum and maximum age of fetus was 3 and 29 weeks, respectively and the minimum and maximum age of the mothers were 16 and 47 years, respectively. Most of the participants had under high school education (64.5%) and more than 94% were housewives. The most common reasons for abortion were fetal abnormalities (70.8%) and then maternal complications (29.25%). Also, 82.7% of the cases (354 person) were eligible for abortion, from which, 81% of the permits were issued due to fetal abnormalities and 19% of them were issued because of maternal complications. Meanwhile, fetal anencephaly (30.3%), fetal hydrops (15.3%), major thalassemia (7.6%), hydrocephalus (6.9%) and cystic hygroma (6.9%) were the most common fetal-related causes of obortion, respectively (Table 1). Furthermore, the most common maternal-related reasons for abortion included; cardiovascular diseases and uncontrolled hypertension (9.3%) (Table 2). Among 428 applications for abortion permit, 74 cases (17.3%) could not get the permit, which were mainly due to maternal disease that lacked indication (39.2%), the use of medication by mother (20.2%) and the high fetal age (17.6%) (Table 3).

**Table 1:** Frequent causes of fetal and maternal issuing permits for therapeutic abortion

Maternal causes	n (%)	Fetal causes	n (%)
Cardiovascular diseases and uncontrolled hypertension	40 (9.3)	Cerebral abnormalities	225 (52.6)
Teratogenic medications	23 (5.4)	Genetic abnormalities	34 (7.9)
Neurological diseases	14 (3.3)	Gastrointestinal abnormalities	14 (3.3)
kidney disease	11 (2.6)	Musculoskeletal abnormalities	5 (1.2)
Cancer	11 (2.6)	Advanced kidney disease	5 (1.2)
Psychological disorders	9 (2.1)	Twin problems	2 (1.2)
Autoimmune diseases	5 (1.2)	Other	18 (4.2)
X-Ray exposure	3 (0.7)	Total	303 (70.8)
Hematological disorders	2 (0.4)		
Viral diseases	2 (0.4)		
Other	5 (1.2)		
Total	125 (29.2)

**Table 2:** Frequent causes of not issuing permits for therapeutic abortion

Causes	n (%)
Maternal disease lacking indication	29 (39.2)
Taking medicine by mother	515 (20.2)
High fetal age	13 (17.6)
Fetal disease lacking indication	8 (10.8)
Pregnancy Conditions	3 (4.1)
Mother with infectious diseases	2 (2.7)
Other	4 (5.4)
Total	74 (100)

**Table 3:** Frequent causes of not issuing permits for therapeutic abortion

Causes	n (%)
Maternal disease that lack indication	29 (39.2)
Taking medicine by mother	15 (20.2)
High fetal age	13 (17.6)
Fetal disease that lack indication	8 (10.8)
Pregnancy conditions	3 (4.1)
Mother with infectious diseases	2 (2.7)
Other	4 (5.4)
Total	74 (100)

### Discussion

Results of the present study showed that, more than 82% of the requests for abortion permit were successful. The study of Rostamnejad et al. showed that 55 (70.5%) out of 78 pregnant women applying for abortion permit received the permission and 23 of them (29.5%) did not get the permit [14]. The study of Naeeji et al. also showed that, the abortion permit was issued for 71.8% of those applying to the department of forensic medicine in Tehran and the requests of 28.2% of them were not approved [17]. Furthermore, Souri showed that 70% of the requests for abortion permit were approved [18]. Results of the aforementioned studies indicated that more than two-third of the applications for abortion permit received the permission which indicates the removal of obstacles for therapeutic abortion. This also shows that, a significant step has been taken towards the promotion of women’s health.

In our study, fetal and maternal problems accounted for 81% and 19% of abortion indications, respectively. The study of Dadipoor et al. showed that 87.5% of the applications for abortion permit were due to fetal problems and 12.5% of them were related to maternal complications [19]. Naeeji et al. also found that 80% of the requests for abortion permit were related to fetal problems [17]. Furthermore, the study of Maleki et al. revealed that 78.8% of the therapeutic abortions in 2007 had fetal-related indications [20]. In the study of Rostamnejad [14] 87.2% and in the study of Souri [18] 88% of abortion were due to fetal abnormalities. The study of Ghadipasha [15] and Sayedoshohadaie [21] indicated that fetal problem was the main cause of therapeutic abortions. The results of these studies are in line with the findings of the present study. This is most probably due to increased prenatal care and accuracy of diagnosis such as ultrasound examination and early detection of fetal abnormalities. The physicians have also been increasing their knowledge on indications for therapeutic abortions and refer more mothers for abortion due to fetal problems.

The result showed that, the most important reason for fetal abortion was related to brain disorders, particularly anencephaly (30.3%). However, the study of Dadipoor et al. found thalassemia as the most common cause of abortion (41.5%) followed by anencephaly (18.2%) [19]. Anencephaly was also found to be the cause of 43% of the abortions in the study of Maleki et al. [20]. Result of the Souri’s study showed that 34% of the abortions were due to the fetal skull and brain disorders [18]. This issue requires more attention of the officials to provide proper educational programs for mothers before marriage and pregnancy in order to prevent neural defect tubes. Furthermore, it seems that adopting health strategies such as preventing individuals with minor thalassemia to get married to each other and providing pre-marriage advice for couples would be effective in reducing the ratio of therapeutic abortions caused by major thalassemia.

Results of the present study showed that, the most common maternal reasons for successful abortion permit were related to cardiovascular diseases and uncontrolled hypertension. The study of Souri found the maternal cardiovascular disease and Hematologic disorders as the most common causes of therapeutic abortion [18]. The study of Dadipoor et al. also reported the maternal cardiovascular disease as the most common reason for abortion [19], and the study of Schiavon et al. reported the maternal hypertension as the most common cause of abortion [22]. In our opinion, cardiologists and gynecologists should help the provision of necessary and preventive trainings for this group of mothers to prevent such consequences.

Our results showed that 17.3% of the requests for abortions were not approved, and the most common reasons for that included maternal problems that lacked indication, the use of medication by mothers and the high fetal age. Souri also found that, making application after the legal time, the use of medication by mothers and maternal problems that lack indication are the most common reasons for the rejection of abortion request [18]. According to the law, maternal problems or use of medication by mother without confirmed fetal abnormalities or endangering mother’s life have no indication for therapeutic abortion [1]. None of these requests were approved in this study.

As a conclusion, most of the 428 pregnant women, who applied to the department of forensic medicine to get permission for therapeutic abortion, could get the permission. The fetal problem was the main reason for issuing permits. The most common fetal-related causes included; anencephaly and fetal hydrops. The most common maternal-related causes of issuing abortion permit included cardiovascular diseases and uncontrolled hypertension. In general, it can be claimed that, increased knowledge of physicians and clinical personnel on indications of therapeutic abortions and related regulations would lead to the implementation of strategies which prevent void referrals to the department of forensic medicine and a better execution of therapeutic abortion law. For a better execution of this law, the physicians and clinical staff should not consider the probability of risks for mother or fetus as indications for therapeutic abortion and also should perform comprehensive examination so that, mothers could be introduced to legal abortion centers in case of certain diagnosis.

## Limitation

It is almost impossible to compare the findings of present study with the studies conducted in other countries due to differences in therapeutic abortion legislations.

## Data Availability

Data and material are available by contacting to corresponding author. In order to access the raw data, it is necessary to obtain the permission of University Research Vice-Chancellor.
